# Topological and Functional Diversity of Gut Microbiota Metabolism Across the Human Lifespan

**DOI:** 10.3390/metabo16020140

**Published:** 2026-02-19

**Authors:** Benjamí Pérez-Rocher, Mariana Reyes-Prieto, Susana Ruiz-Ruiz, Pere Palmer-Rodríguez, José Aurelio Castro, Andrés Moya, Mercè Llabrés-Segura

**Affiliations:** 1 Institute for Integrative Systems Biology (I2SysBio), University of València and CSIC, 46980 València, Spain; andres.moya@uv.es; 2 Sequencing and Bioinformatics Service, Foundation for the Promotion of Sanitary and Biomedical Research (FISABIO), 46010 València, Spain; bertha.reyes@uv.es; 3 Genomics and Health Area, Foundation for the Promotion of Sanitary and Biomedical Research (FISABIO), 46010 València, Spain; susana.ruiz@fisabio.es; 4 Biomedical Research Center Network of Epidemiology and Public Health (ciberESP), C/Monforte de Lemos 3-5. Pabellón 11., 28029 Madrid, Spain; 5 Department of Mathematics and Computer Science, University of the Balearic Islands, 07122 Palma de Mallorca, Spain; pere.palmer@uib.es (P.P.-R.); merce.llabres@uib.es (M.L.-S.); 6 Department of Biology, University of the Balearic Islands, 07122 Palma de Mallorca, Spain; jose.castro@uib.es

**Keywords:** gut microbiota, metabolic networks, metabolic DAGs, reaction graphs, functional profiling

## Abstract

**Background:** The human gut microbiota plays a central role in host physiology by influencing digestion, immune function, and metabolism. Characterizing age-associated differences in the organization of microbial metabolism may provide insights into functional variation in the gut microbiome across the human lifespan. **Methods:** Gut microbiota metabolic organization was analyzed in a cohort of 30 individuals spanning three age groups (infants, adults, and elderly individuals) and comprising 156 stool samples. Community metabolic networks were reconstructed using the metabolic Directed Acyclic Graph (m-DAG) framework derived from KEGG Ortholog annotations. Network topology was characterized to assess whether the resulting networks conform to previously described global structural patterns and to examine age-associated variability. Pairwise m-DAG dissimilarities were computed, and hierarchical clustering was applied to evaluate similarities among samples. **Results:** All samples revealed a conserved global network organization, alongside marked variability in specific structural features. Hierarchical clustering did not strictly reflect chronological age. A homogeneous cluster composed exclusively of adult samples was identified, whereas elderly samples were distributed across two clusters, one grouping with adults and the other with infants. Exploratory discriminative analyses identified functional reactions contributing to the separation between the adult cluster and the remaining samples, indicating age-associated differences in metabolic network organization. **Conclusions:** Gut microbiota metabolic networks in adults tend to exhibit lower redundancy and structural complexity, whereas those in infant and elderly samples display more heterogeneous network configurations. This network-based analysis provides a functional perspective on age-associated variation in gut microbiota metabolism and offers a framework for future integrative studies.

## 1. Introduction

The human gut microbiota plays a central role in host physiology, contributing to nutrient metabolism, immune system development, xenobiotic processing, and the overall health of the host [1,2]. The term microbiota refers to the community of microorganisms inhabiting the host, whereas the microbiome encompasses their collective genomes and functional potential, including metabolic capabilities and interactions with the host [3]. Inter-individual variability in microbial communities reflects the influence of diet, lifestyle, geography, and early-life exposures [4,5].

Alterations in gut microbiota composition and function have been associated with a wide range of human diseases. In gastrointestinal disorders such as inflammatory bowel disease, consistent shifts in microbial structure and metabolic activity have been reported [6,7]. Beyond the gut, perturbations of the microbiome have been linked to metabolic and cardiometabolic conditions, including obesity, type 2 diabetes, non-alcoholic fatty liver disease, and cardiovascular disease, highlighting the close relationship between microbial metabolism and host homeostasis [2,8,9]. In addition, accumulating evidence supports bidirectional interactions between the gut microbiota and the nervous system through the gut–brain axis, mediated in part by microbially derived metabolites such as short-chain fatty acids [10,11].

Despite these associations, defining the characteristics of the gut microbiota under homeostatic conditions (i.e.*,* a “healthy” gut microbiota) remains challenging. Concepts such as microbial dysbiosis lack a universal reference baseline, and microbial configurations compatible with health appear to be context-dependent and dynamic, shaped by factors including age, diet, environment, host genetics, and immune status [12,13]. Among these factors, age is known to strongly influence both microbial composition and functional potential throughout the human lifespan [14]. Thus, characterizing the topology of these metabolic networks may reveal latent functional patterns associated with age.

Studies of the gut microbiota typically address two complementary dimensions: taxonomic composition and functional potential, encompassing gene content, enzymatic capabilities, and metabolic outputs [15]. While taxonomic profiling provides insights into community structure, functional characterization requires analytical frameworks capable of capturing enzyme-level contributions and the network organization underlying metabolic processes [16,17]. In this context, enzymatic reactions form interconnected networks whose structure constrains the range of metabolites that can be produced and exchanged within the microbial community.

Recent advances in large-scale sequencing technologies and curated metabolic databases (e.g., KEGG and MetaCyc) have enabled the reconstruction of community-scale metabolic networks from functional annotations [18,19,20].

In this study, we apply the metabolic Directed Acyclic Graph (m-DAG) framework to metagenomic gut microbiota data to provide an exploratory and descriptive analysis of community-level metabolic networks across three age groups: infants, adults, and elderly individuals. Rather than aiming to establish causal mechanisms or taxonomic drivers, our objective is to characterize the topological organization of these networks, assess their variability within and between age groups, and examine how similarities and differences in metabolic network structure relate to age-associated patterns. This network-based approach provides a functional perspective on gut microbiota metabolism and establishes a foundation for future integrative and mechanistic studies.

## 2. Materials and Methods

### 2.1. Cohort Recruitment and Sampling

The cohort consisted of thirty healthy volunteers from the Valencian Community (Spain): ten infants (I1 to I10) aged 2–5 years (mean age 3.9 ± 1.5 years), ten adults (A1 to A10), aged 27–44 years (mean age 35.4 ± 6.6 years), and ten elderly individuals (E1 to E10) aged 69–81 years (mean age 74.7 ± 4.0 years). For each participant, up to 8 fecal samples were collected over 2 years following standardized instructions provided by the research team. After participant withdrawal and exclusion of one individual (E08) due to a cancer diagnosis, a total of 219 samples were initially available. However, samples with insufficient sequencing depth were excluded from downstream analyses, resulting in a final dataset of 156 samples, which served as the basis for the study. Subsequently, some were identified as outliers, as detailed in Section 3.

All participants completed a questionnaire collecting information on diet, general health, medical history, and use of antibiotics and other medications. Individuals who received antibiotic treatment during the sampling period waited at least two weeks after completing treatment before collecting subsequent samples. All personal identifiers were anonymized.

Although samples were collected at different time points, they were not analyzed as a temporal series. Instead, samples from the same individual were treated as repeated measurements capturing intra-individual variability rather than as time-ordered observations.

### 2.2. DNA and RNA Purification

Fecal samples were resuspended in a 50% RNAlater^TM^ (Invitrogen, Waltham, MA, USA) and phosphate-buffered saline (PBS) solution and centrifuged to remove particulate debris. The supernatants were aliquoted. Total DNA was extracted from a 500 µL aliquot of the pellet using the MagNA Pure LC DNA Isolation kit III (Bacteria, Fungi) (Roche Diagnostics, Mannheim, Germany) and the MagNA Pure LC Robot (Roche Diagnostics, Mannheim, Germany).

DNA and RNA concentrations were quantified using the Qubit^TM^ dsDNA HS Assay Kit and Qubit™ RNA HS Assay Kit, respectively, on a Qubit^TM^ 4.0 fluorometer Thermo Fisher Scientific, Waltham, MA, USA.

### 2.3. Metagenome Sequencing

Metagenomic libraries were prepared from total DNA using the Nextera XT DNA Library Preparation kit (Illumina, Inc., San Diego, CA, USA). DNA fragmentation and adapter ligation were performed using Nextera XT transposase, followed by limited-cycle PCR amplification. PCR products were purified using AMPure XP beads (Beckman Coulter). Libraries were quantified using the Qubit™ DNA HS Assay Kit (Thermo Fisher Scientific), and an equimolar library pool was prepared. The sequencing was performed on a MiSeq sequencer (Illumina), which produced paired-end reads of 2 × 300 bp.

### 2.4. Sequence Processing and Functional Annotation

Raw shotgun reads were quality-filtered and trimmed using Fastp v0.21.0 [21]. Adapter sequences were auto-detected and removed. Quality trimming was performed using a sliding window approach (window size 4, quality threshold of 15), with leading and trailing bases trimmed at a quality score of 20, and reads truncated at 290 bp. Reads containing ambiguous bases (N) or shorter than 60 bp were discarded.

Contaminant sequences, including Phix (GCF_000819615.1) and host-derived reads (human genome GRCh38.p13) were removed using Bowtie2 v2.4.2 [22] and Samtools v1.10 [23]. Regions of the human reference genome corresponding to known contaminants were masked using Bedtools v2.29.2 [24]. Quality control was assessed at multiple stages using FastQC v0.11.9 [25] and summarized with MultiQC v1.9 [26]. Samples with fewer than 100,000 reads were excluded from further analysis, leading to the removal of all samples corresponding to time points T2 and T3, as well as sample E05T8.

Functional annotation was performed using the SqueezeMeta pipeline [27] with KEGG database annotations. Abundances of KEGG Orthology (KO) groups were obtained and used as input for the reconstruction of reaction graphs and metabolic Directed Acyclic Graphs (m-DAGs).

### 2.5. Metabolic Network Construction: Reaction Graphs and m-DAGs

The definitions of Reaction Graph (RG) and metabolic Directed Acyclic Graph (m-DAG) used in this study follow those introduced in [28] and are briefly summarized here for clarity and completeness. The RG representation of a metabolic network is a directed graph whose nodes are the set of chemical reactions present in the metabolism. Its set of arcs is defined as follows: There is an arc from a reaction *R* to *R*′ if, and only if, there is at least one metabolite produced by *R* that *R*′ consumes. The nodes are labeled with the KEGG identifiers of the corresponding reaction, and the edges are not. Reversible reactions are modeled using two nodes, one for the forward reaction and the other for the backward reaction. Reversible reactions are labeled by the label of the forward reaction, followed by “rev”.

A directed acyclic graph (DAG) is a directed graph that contains no cycles. In a directed graph *G*, a path from a node *u* to a node *v* is a sequence of nodes {*u*_0_, *u*_1_, ..., *uₖ*} such that *u*_0_ = *u*, *uₖ* = *v*, and for every *i* = 0, ..., *k* − 1, there exists a directed edge (arc) (*u*_i_, *u_i_*_+1_) in *G*. Two nodes *u* and *v* are said to be biconnected if there is a path from *u* to *v*, and also a path from *v* to *u*. A strongly connected component of a directed graph *G* is a maximal subgraph in which every pair of nodes is biconnected [29,30]. Since biconnectivity is an equivalence relation, the set of strongly connected components forms a partition of the node set of *G*.

By contracting each strongly connected component into a single vertex, we obtain the graph’s condensation, which is a DAG. There is an arc from a node *sᵢ* to a node *sⱼ* in the condensation of *G* if and only if there is at least one arc in *G* from a node *u* in *sᵢ* to a node *v* in *sⱼ*. Given a reaction graph *G_R_*, its condensation is a DAG. Notably, by construction, each connected pair of nodes in the original graph either collapses into a single node (if they belong to the same strongly connected component) or remains as a connected pair in the condensation graph (if they belong to different strongly connected components). Therefore, the condensation graph preserves the number of connected components of the original graph.

We refer to the strongly connected components in *G_R_* as metabolic building blocks (MBBs), and we call the condensation of *G_R_* the metabolic DAG (m-DAG). An MBB consisting of a single reaction is considered essential in the m-DAG if its removal increases the number of connected components in the graph. All reaction networks and their corresponding m-DAG representations from our dataset were generated using the metaDAG web tool [20]. Reaction graphs and m-DAGs were constructed from sample-specific lists of KEGG Orthologs (KOs): each KO was mapped to its corresponding enzyme, and all reactions catalyzed by these enzymes were retrieved and assembled into the corresponding reaction graph.

### 2.6. Pairwise Similarity of m-DAGs and Clustering Analysis

As introduced in [28], the Munkres similarity for comparing m-DAGs is based on the maximum-weighted bipartite matching algorithm (also known as the Hungarian or Munkres algorithm). It is defined as follows:

Step 1: Similarity between MBBs:

- A complete bipartite graph is constructed between the reactions in two MBBs (MBB_1_ and MBB_2_).
- Each edge between reactions is weighted using a predefined reaction similarity score.
- The maximum weighted bipartite matching algorithm is applied to find the best pairing between reactions in MBB_1_ and MBB_2_.
- The similarity score S_mbb_(MBB_1_, MBB_2_) is calculated as the sum of the matched reaction scores divided by the size of the larger MBB.

Step 2: Similarity between m-DAGs:

- Another complete bipartite graph is constructed, this time between the MBBs of two m-DAGs (mD1 and mD2), with edge weights set to the MBB similarity scores computed in Step 1.
- The maximum weighted bipartite matching is applied again to find the best mapping between MBBs.
- The final similarity score Sim(mD1, mD2) is obtained by summing the matched MBB similarities and dividing by the number of MBBs in the larger m-DAG.

The computed similarity was transformed into a dissimilarity measure by applying the function $\sqrt{\left(1-{similarity}^{2}\right)}$. Hereafter, this transformed quantity is referred to as the Munkres dissimilarity.

To analyze the structural properties of the metabolic networks associated with each age group, we computed topological features from their corresponding metabolic DAGs. These DAGs were analyzed using the igraph package in R [31,32], which provides a comprehensive suite of functions for network analysis and the extraction of a range of topological descriptors. Also, pairwise dissimilarities between m-DAGs were quantified using the Munkres-based dissimilarity, derived from the corresponding graph similarity scores. The resulting dissimilarity matrix was used as input for hierarchical clustering, performed using an agglomerative approach with complete linkage. The optimal number of clusters was determined using the Elbow method [33] and the Silhouette method [34], and the resulting clusters were used for downstream analyses. Cluster stability was assessed using multiscale bootstrap resampling implemented in the pvclust R package 2.2-0 [35]. Because clustering was based on a dissimilarity matrix, the Munkres dissimilarities were first embedded into a Euclidean space using classical multidimensional scaling (MDS). Hierarchical clustering with complete linkage and Euclidean distance was then applied to the resulting coordinates, and cluster robustness was quantified using approximately unbiased (AU) values obtained from 1000 bootstrap replicates.

### 2.7. Discriminative Analysis Discriminant Analysis Procedure

Sparse Partial Least Squares Discriminant Analysis (sPLS-DA) was performed using the splsda( ) function from the mixOmics R package [36,37]. sPLS-DA was used as an exploratory discriminative method to visualize group-associated variation rather than as a predictive classifier. Accordingly, no classification performance metrics were computed, and results were interpreted in conjunction with unsupervised and graph-based analyses.

Models were fitted with two latent components (ncomp = 2), and sparsity was enforced by selecting a limited number of variables per component (keepX = c(50, 30)). These parameters were chosen to summarize discriminative information in a low-dimensional space while retaining only the most informative variables on each component, thereby constraining model complexity and enhancing interpretability and stability. All pairwise group comparisons were performed independently. Discriminant MBBs were selected based on their contribution to group separation, quantified by the values.var metric. Variables with absolute values ≥ 95% of the maximum absolute value within each component were retained, and their sign was used to assign group association.

Functional interpretation of discriminant MBBs was performed by retrieving the associated metabolic reactions using the KEGGREST R package [38], enabling pathway-level interpretation of the discriminant features.

Other R libraries were used for specific analyses, including ggplot2 [39] for data visualization and ppclust [40] and cluster [41] for cluster analysis.

## 3. Results

In this section, we analyze gut microbiota metabolic networks represented as m-DAGs. We first describe their topological properties across age groups, focusing on network size and connectivity. We then compare metabolic networks using pairwise dissimilarity measures and hierarchical clustering to identify structural similarities among samples. Finally, we apply discriminative analyses to identify metabolic building blocks and reactions that contribute to the differences observed among the resulting clusters.

### 3.1. Topological Analysis

**Figure 1:**
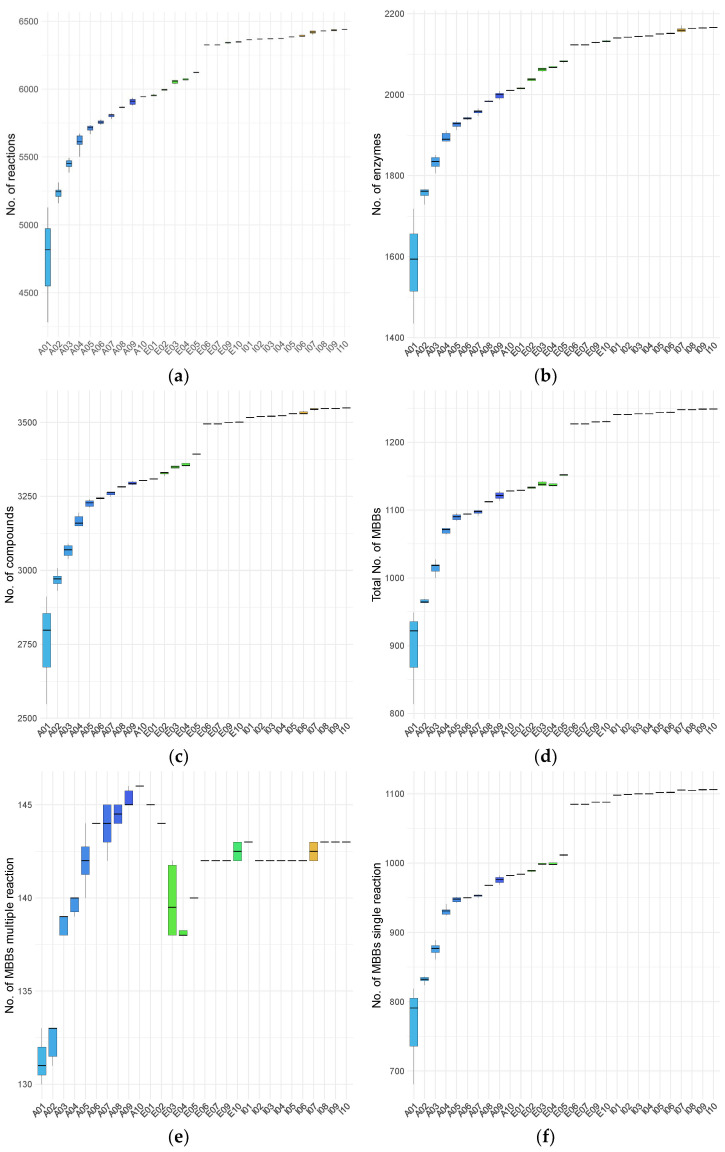
Network size properties of metabolic networks across individuals and age groups. (**a**) Number of reactions; (**b**) number of enzymes; (**c**) number of compounds; (**d**) total number of MBBs; (**e**) number of MBBs containing more than one reaction; (**f**) number of single-reaction MBBs. Colors indicate age group: adults (blue), elderly individuals (green), and infants (red-orange).

To characterize age-associated differences in metabolic network organization, we examined the topological properties of sample-specific m-DAGs. As established in [20], the topology of m-DAGs consists of a large connected component followed by numerous tiny connected components and isolated nodes. Within the large connected component, there is a node (an MBB) that integrates a vast number of reactions from essential pathways, including carbohydrate metabolism, energy metabolism, and nucleotide metabolism. Our analyses focused on network size, connectivity, and structural descriptors of the largest connected component, allowing comparison of global and local network features across infants, adults, and elderly individuals. Regarding the network size, Figure 1 summarizes the numbers of reactions, enzymes, and compounds in the constructed RGs, as well as the number of MBBs (nodes) in the corresponding m-DAGs, color-coded by age group. Apparent differences in network size are observed across samples and age groups.

Overall, adult samples exhibit the lowest numbers of enzymes, reactions, and total MBBs, whereas infant samples show the highest values for these properties. Elderly individuals display intermediate profiles, with some individuals showing values slightly above those observed in adults and clearly below those of children, while others exhibit values comparable to those of the pediatric group. Notably, adults show substantial intra-group variability in the number of MBBs containing more than one reaction, spanning nearly the full range observed across the dataset. Across all topological properties, at least one adult individual (A01) consistently exhibits the broadest range of variation.

**Figure 2:**
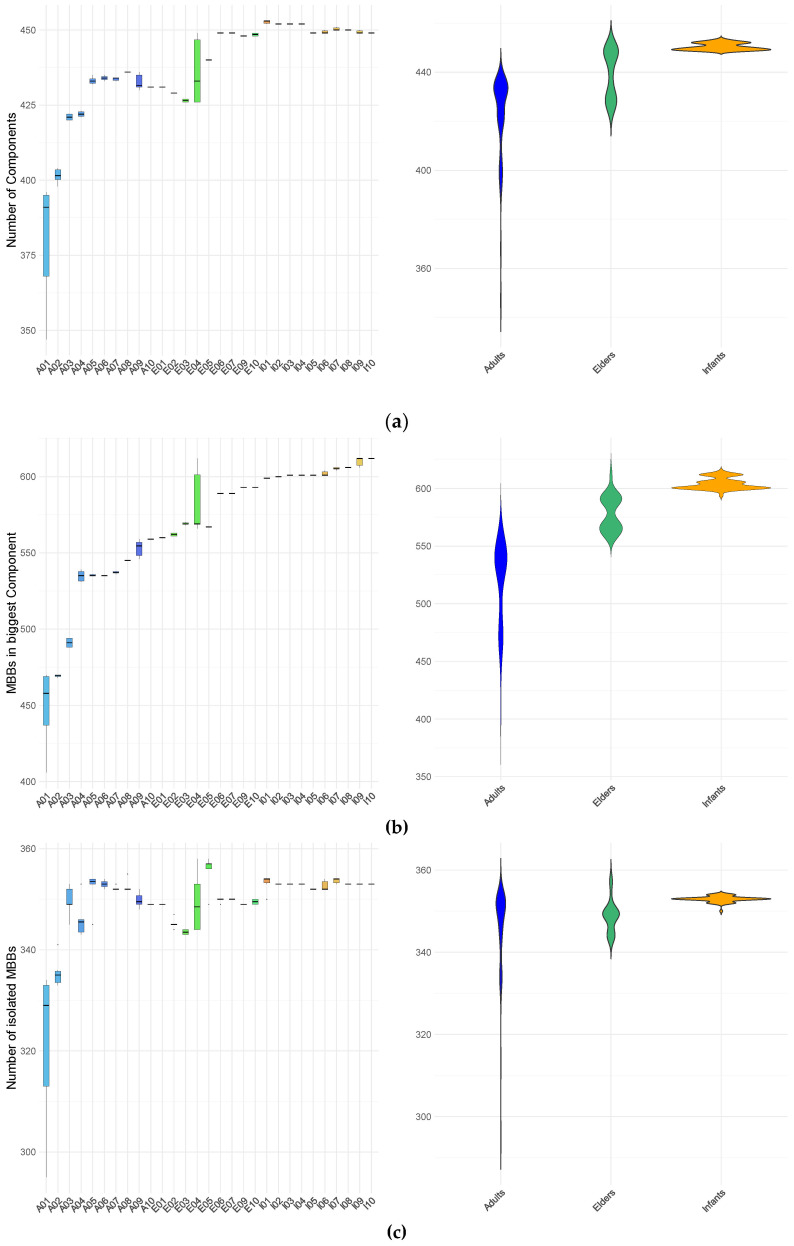
Connectivity properties of m-DAGs across samples and age groups. (**a**) Number of connected components per m-DAG; (**b**) size (expressed as the number of MBBs) of the largest connected component; (**c**) number of isolated MBBs. Results are shown by individual (left panels) and by age group (right panels). Colors indicate age group: adults (blue), elderly individuals (green), and infants (red–yellow).

Regarding network connectivity, Figure 2 summarizes the number of connected components in each m-DAG, their node counts (MBBs), and the number of isolated nodes. Across the entire cohort, a consistent structural pattern is observed: a single large connected component, a large number of small components containing only a few nodes, and numerous isolated nodes. This organization closely resembles the previously reported structure of m-DAGs across the four kingdoms (Animals, Plants, Fungi, and Protists) in [20].

At the level of metabolic building blocks (MBBs), each network contains one exceptionally large MBB, averaging approximately 4497 reactions, alongside a long tail of MBBs with substantially fewer reactions. The number of these smaller MBBs varies widely across samples, ranging from 413 in sample A10T4 to 1254 in sample I07T6, with sizes spanning from ten reactions down to single-reaction MBBs. Network size (number of MBBs), the total number of connected components, and the component size distribution for all samples are summarized in Table S1.

**Figure 3:**
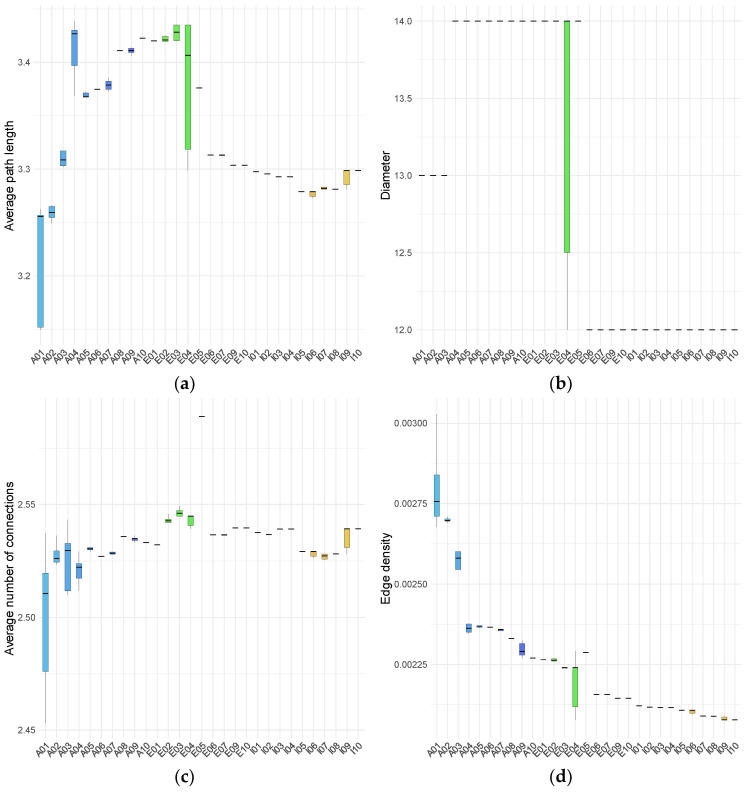
Topological properties of the largest connected component of m-DAGs across all samples, grouped by age. (**a**) average path length; (**b**) diameter; (**c**) average node degree; (**d**) edge density. Box plot colors indicate individual subjects within each age group. Colors denote age group: adults (blue scale), elderly individuals (green scale), and infants (red–yellow scale).

For the larger connected component, we quantified several topological descriptors, including the number of nodes, diameter, average number of connections (also known as node degree), the average path length, and edge density. Network diameter was defined as the maximum shortest path between any two nodes, and average node degree was calculated as the mean number of connections per node. These properties are summarized in Figure 3 and Table S2.

The topological properties of the largest connected component show pronounced differences between infants and the adult and elderly groups. Although the average node degree does not differ markedly across age groups, the dispersion of values is substantially larger for specific adult and elderly individuals. In particular, one adult (A01) and one elderly participant (E04) display considerable intra-individual variability across all examined properties, including the number of nodes (corresponding to the number of MBBs), edge density, average path length, average node degree, and diameter. This variability is primarily driven by pronounced differences among specific samples from these individuals, which deviate from the remaining samples. Consistently, a multivariate analysis integrating m-DAG topological properties and essential-node descriptors identified A01T1, A01T4, and A01T5 as global multivariate outliers. Features were standardized and projected onto a principal component space retaining 95% of the total variance, and squared Mahalanobis distances were evaluated using a chi-square distribution with false discovery rate correction.

Infant samples generally display narrow ranges across the m-DAG properties studied, except for average node degree. There are minimal variations between samples from different infants, or at least these variations are less pronounced than those observed in adults and elders. Clear differences among age groups are evident: the number of nodes in the largest connected component is highest in infants, lowest in adults, and intermediate in elders.

Analysis of diameter values reveals that infants consistently exhibit low invariant diameters across individuals and samples. In contrast, adults and elderly groups exhibit larger diameters with noticeable inter- and intra- individual variability. Within the adult group, three individuals show samples with intermediate diameter values (13), and one individual displays a single sample at the maximum observed value (14). Among the elders, four individuals have the lowest diameter values, with one displaying substantial intra-individual variation (ranging from 12 to 14) and another reaching the highest maximum value (14), while another has the minimum value (12).

A similar pattern is observed for average path length: infant samples demonstrate low intra- and inter-individual variability, whereas elderly samples display higher average values, and adult samples exhibit the widest range of variation. Edge density is highest in adults, with elevated values observed in samples from at least four individuals. Elderly participants exhibit marked inter-individual heterogeneity: five display edge densities at the lower end of the adult range, while the remaining five show values comparable to those observed in infants. Notably, only one elderly individual demonstrates a wide range of intra-individual variation. Infant samples consistently exhibit the lowest edge density, with minimal variation within and between individuals. As edge density reflects the proportion of realized connections among nodes, these results indicate a more tightly interconnected network structure in adults.

Overall, infants exhibit low intra- and inter-individual variability across all analyzed parameters, in contrast to the broader variability observed in adult and elderly individuals. Within the elderly group, four individuals (E06, E07, E09, and E10) show profiles more similar to those of infants. In contrast, the rest align more closely with the lower values observed in adults, particularly in terms of the number of nodes, diameter, average path length, and edge density.

**Figure 4:**
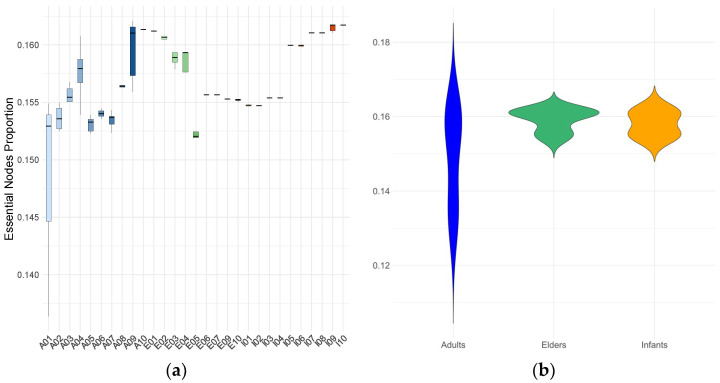
Proportion of essential MBBs in the m-DAGs for each individual (**a**) and age group (**b**). Box plot colors indicate individual subjects within each age group. Colors denote age group: adults (blue or blue scale), elderly individuals (green or green scale), and infants (orange or red-orange scale).

To conclude the topological analysis, we examined the essential (critical) nodes of all m-DAGs. Essential nodes are those whose removal disrupts graph connectivity (see Methods). As shown in Figure 4, the global range of the proportion of essential nodes is broader, and the average is lower in adults, primarily due to extreme values observed in individual A01. Most adults (except A08 and A10), as well as several elders (E02, E03, E04, and E05), display substantial variability in the proportions of essential metabolic building blocks (MBBs).

In contrast, infant samples show low variability in the proportion of essential MBBs, although a subset of individuals (I01, I02, I03, and I04) consistently present lower values than their peers.

### 3.2. Munkres Dissimilarity on m-DAGs

**Figure 5:**
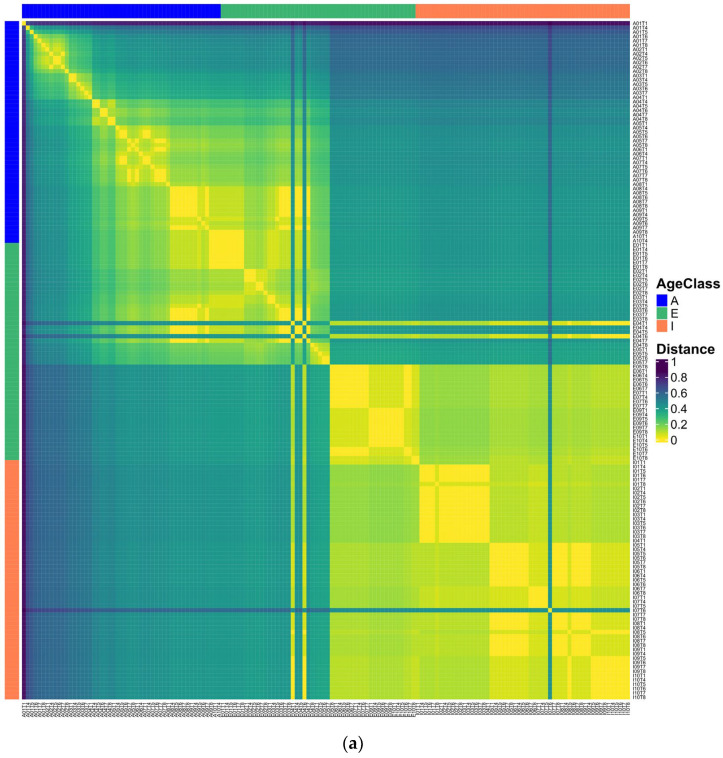

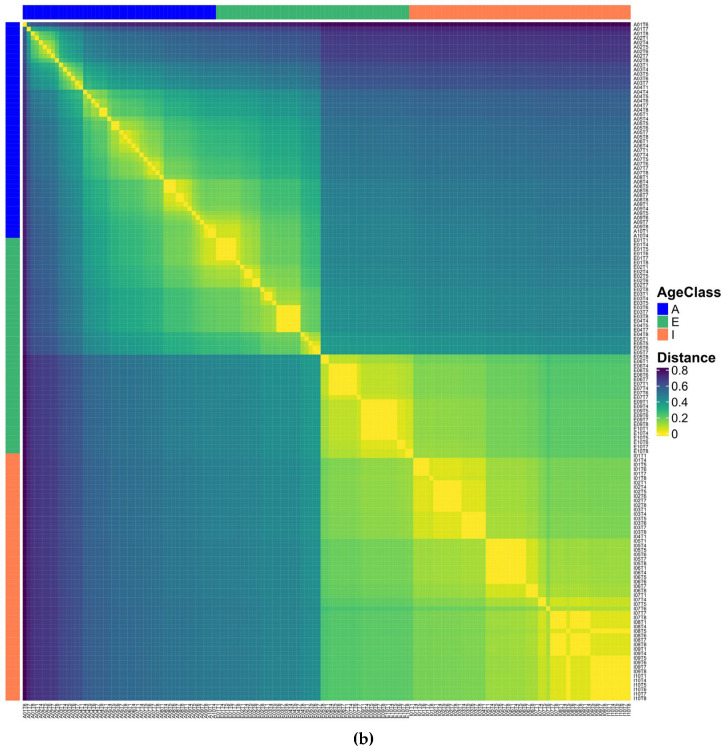
Heatmaps of pairwise Munkres dissimilarity between m-DAGs. (**a**) Dissimilarity matrix including all samples; (**b**) dissimilarity matrix after exclusion of outlier samples. Sample labels indicate age group: adults (A), infants (I), and elderly individuals.

We calculated the Munkres similarity of all m-DAGs as defined in [28] and obtained the corresponding Munkres dissimilarity measure (see Section 2). A heatmap of the Munkres dissimilarity matrix across all m-DAGs was used to visualize pairwise structural differences between metabolic networks (Figure 5).

In this representation, the samples E04T1 and E04T6 stand out for their high dissimilarity relative to most other m-DAGs, reflecting pronounced structural differences in their reaction graph organization. To prevent highly atypical samples from disproportionately influencing subsequent analyses, these samples were excluded along with A01T1, A01T4, and A01T5, which were previously identified as global multivariate outliers. The Munkres dissimilarity matrix was then recomputed for the remaining m-DAGs and visualized again.

The updated heatmap representation reveals a more homogeneous pattern of pairwise dissimilarities, facilitating the interpretation of global structural relationships among metabolic networks and supporting downstream clustering analyses. Two main clusters are evident: one comprising all the adult samples together with several elderly samples, and another grouping all infant samples with the remaining elders. Furthermore, the heatmap’s diagonal reveals a clear pattern: samples from the same individual are consistently more similar to each other than to those from different individuals in the cohort, indicating higher intra-individual similarity in metabolic network structure.

**Figure 6:**
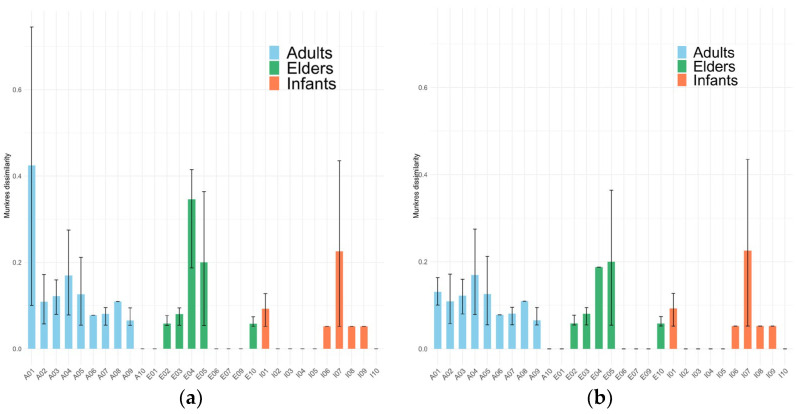
Barplots representing the average of Munkres dissimilarity between samples from each individual studied with outliers (**a**) and without outliers (**b**). Error bars represent the range of distances calculated between intra-individual samples. Note that only one sample from individual I04 is considered, and, in consequence, its average of Munkres distances and error bar is 0. But, I02, I05, and I10 consider six samples each.

To assess metabolic heterogeneity at the individual level, Figure 6 presents the average Munkres dissimilarity calculated between samples from the same individual. As observed in Figure 5a, two elderly samples appear in the first and fourth quadrants of the heatmap, displaying high dissimilarity relative to adult samples and to one elderly subgroup, but low dissimilarity with another elderly subgroup and with infant samples. Additionally, one infant sample appears to be a potential outlier, showing greater dissimilarity than all other samples.

Overall, intra-individual Munkres dissimilarity values are lower in infants and higher in adults and elderly individuals. This pattern suggests that, in infants, the metabolic capacities of the gut microbial community exhibit lower temporal variability than those observed in certain adults (A01, A02, A03, and A04) and elderly individuals (E04, E05). These findings highlight a greater stability of the metabolic network structure in infants compared with older age groups.

**Figure 7:**
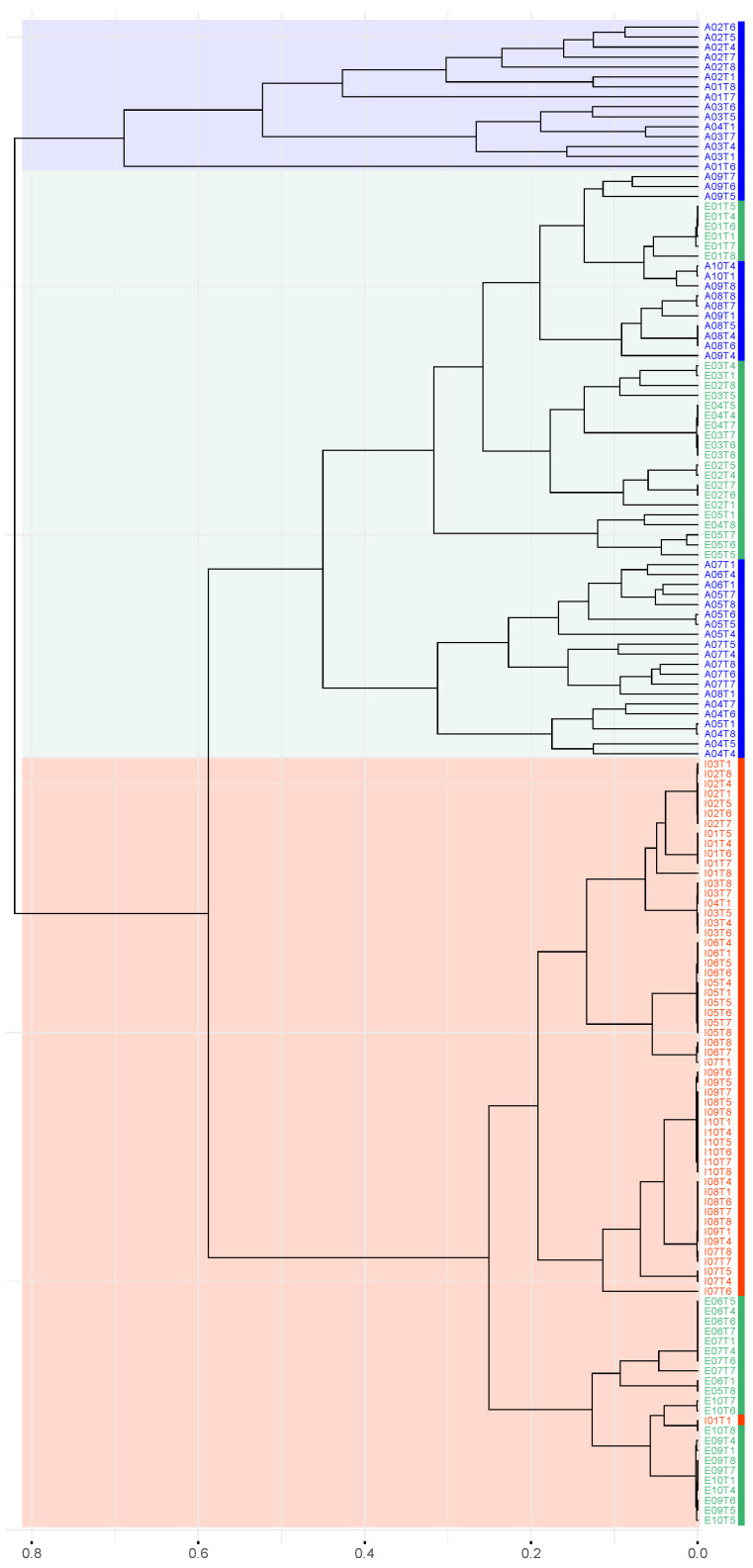
Hierarchical dendrogram based on the Munkres dissimilarity between m-DAGs after exclusion of atypical samples. Vertical color bars adjacent to sample labels indicate age group: adults (blue), elders (green), and infants (red). Shaded regions highlight the major clusters identified.

Additionally, Figure 7 illustrates the hierarchical clustering of samples based on Munkres dissimilarity. The resulting dendrogram reveals that all infant samples, except for one, cluster closely together, indicating low pairwise dissimilarities within this group. Notably, this infant cluster is associated with a subset of samples from elderly individuals and includes the previously mentioned outlier sample from infant I10.

In contrast, samples from several adult individuals form a distinct cluster, clearly separated from the remaining groups, with an outlier adult sample (A01T6) diverging early from the rest. Finally, a third clade comprises samples from the remaining adult and elderly individuals.

The dendrogram does not reveal a clustering structure that strictly follows the three predefined age groups. However, when the dendrogram is cut to obtain three clusters (k = 3), three distinct and interpretable clades can be identified:

- AA (Adults Only): A cluster composed exclusively of adult samples, including all samples from individuals A01, A02, and A03, as well as one sample from A04 (A04T1).
- AE (Adults and Elderly): A mixed cluster comprising samples from both adults and elderly individuals, including adults A05–A10, the remaining samples from A04, and elderly individuals E01–E03, along with four samples each from E04 and E05.
- EI (Elderly and Infants): A cluster primarily composed of elderly and infant samples, including two samples from E04, one sample from E05, and individuals E06, E07, E09, and E10, and all samples from infants I01–I10.

Both the Elbow method [33] and the Silhouette method [34] indicate that the optimal number of clusters is 3. Bootstrap validation recovered the same three major clusters obtained by distance-based hierarchical clustering. All clusters showed strong statistical support, with approximately unbiased (AU) values of 0.99 for AA, 0.99 for AE, and 0.97 for EI. Cluster assignments remained stable under a stringent AU threshold (≥0.9685), indicating high robustness of the identified grouping.

These clusters were designated as AA, AE, and EI in subsequent analyses, and their corresponding samples are summarized in Table 1.

### 3.3. Core and Pan Metabolism Analyses

One of the most important functions of the metaDAG web tool (https://bioinfo.uib.es/metadag/ accessed on 8 February 2026) is the identification of core and pan metabolism in predefined groups of samples. First, we considered the core m-DAG for each individual and for the three clusters defined by Munkres dissimilarity (AA, AE, and EI). The core m-DAG of the entire dataset has 841 MBBs. This core exhibits significant differences compared to the core from the EI group (1223 vs. 841), but shows a very low difference with the AA group (954 vs. 841).

**Figure 8:**
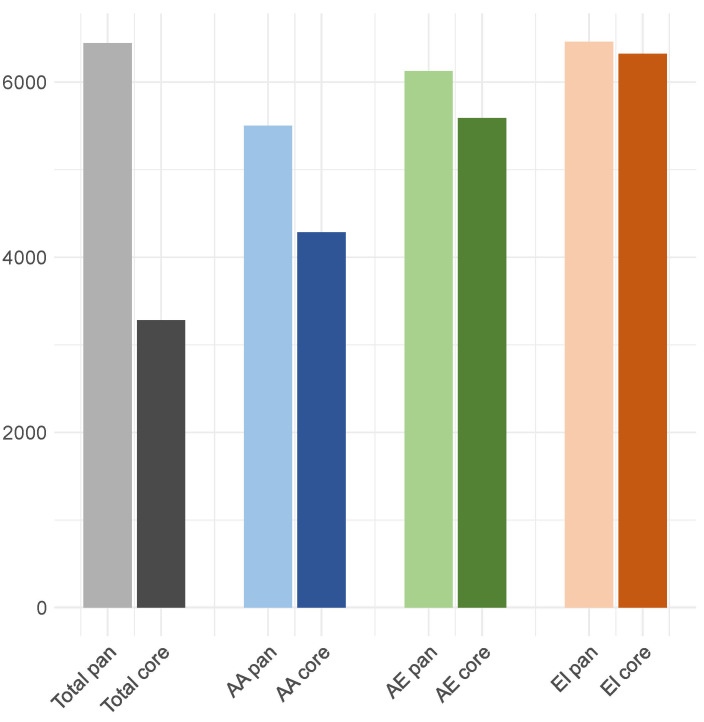
Number of reactions in the core and pan m-DAGs for each cluster and for the total cohort, as defined by Munkres dissimilarity.

The number of essential MBBs in the AA core (111) is markedly lower than in the AE (172) and the EI (191) cores, both in absolute terms and relative to core size (a proportion of 0.1364 versus 0.1607 and 0.1561, respectively). Finally, clear differences are observed in the total number of reactions present in cores, with higher values in the EI group (6322) and lower values in the AA group, and the same in the total core (4283 in both). These results are summarized in Table 2 and Figure 8.

Samples from the infant age group and from the EI cluster show the highest number of reactions (6459 in both cases), encompassing nearly all reactions detected in the cohort. Because the EI cluster includes all infant samples and a subset of elderly samples, the infant group alone captures the full reaction repertoire observed in the dataset. Therefore, the pan-m-DAG derived from the infant group (and equivalently from the EI cluster) corresponds to the pan-metabolic network of the whole cohort. Consistently, all infant samples except one (I01T1) display high similarity to both the infant and cohort-level pan m-DAGs (Table 2 and Table S1).

### 3.4. Discriminant Analysis Performance

As previously indicated, analyzing samples according to their metabolic network similarity rather than predefined age categories provides a more coherent framework for functional comparison. Grouping samples based on Munkres dissimilarity directly reflects the existence of differences in the functional organization of microbial metabolism. Accordingly, we focused on identifying discriminative features among the three clusters defined by this measure (AA, AE, and EI), intending to determine the metabolic building blocks (MBBs) and sets of reactions that differentiate their metabolic capabilities.

**Figure 9:**
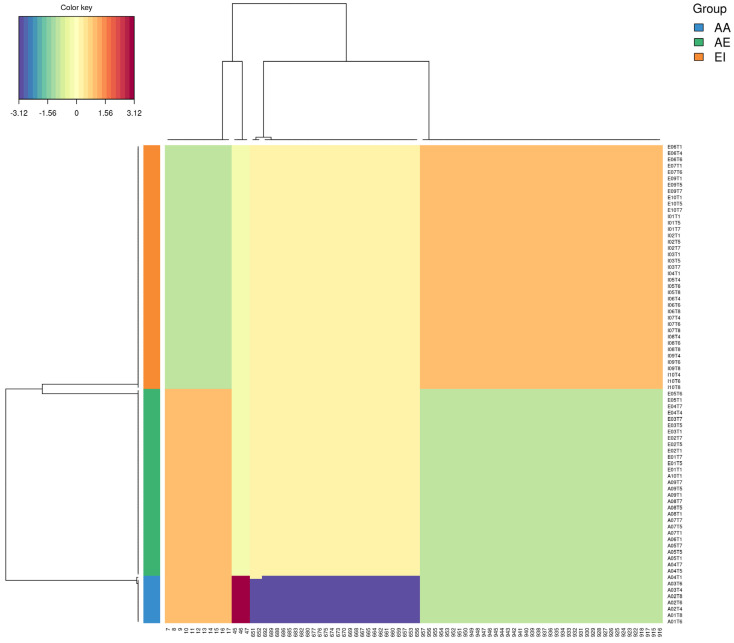
Clustered Image Map (CIM) showing MBBs discriminating among clusters determined by Munkres distance (AA, AE, and EI). The dendrogram on the left represents hierarchical clustering of samples. The horizontal axis denotes discriminant MBBs identified by sparse Partial Least Squares Discriminant Analysis (sPLS-DA).

To achieve this, we applied Sparse Partial Least Squares Discriminant Analysis (sPLS-DA) to identify MBBs contributing to group separation. The sPLS-DA results reveal a substantial number of MBBs that distinctly separate the AA cluster from the other two clusters. Moreover, a substantial set of MBBs also allows for the differentiation between the remaining two clusters (AE and EI), indicating distinct functional patterns within each cluster. These results are shown in Figure 9, which represents a Cluster Image Map (CIM) in the MixOmics framework.

We observe that the AA cluster is strongly characterized by a subset of MBBs that attain the maximum discrimination values. In contrast, discriminative signals are also detected in the remaining two clusters, although with lower magnitudes.

#### 3.4.1. Discriminative Analysis Between Clusters AA and EI

**Figure 10:**
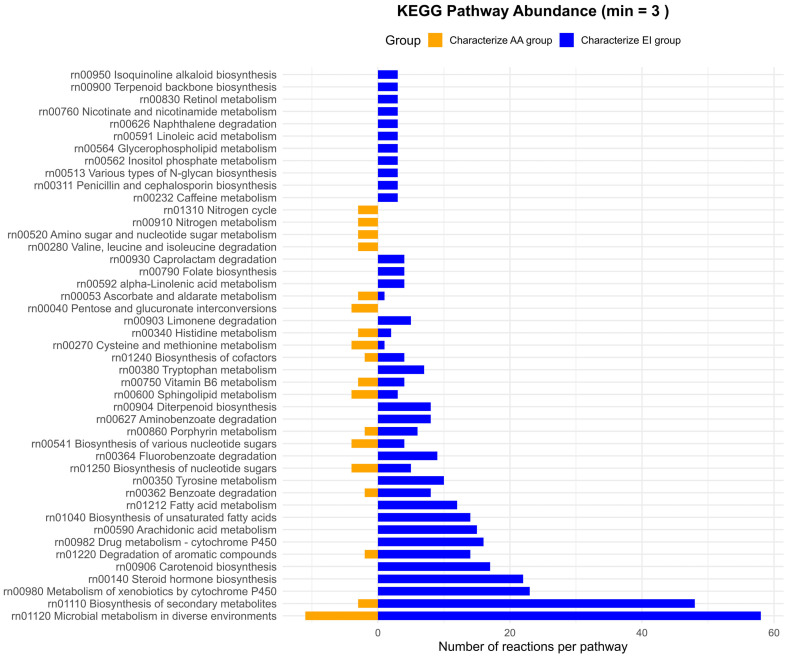
Pathway-level distribution of reactions discriminating between clusters AA and EI. Bar plots show the number of reactions assigned to KEGG pathways that characterize the AA cluster (left, orange) and the EI cluster (right, blue).

As shown in Figure 10, relatively few reactions and pathways specifically characterize the AA cluster compared to the EI cluster, that is, those present in AA but absent in EI. Among these, only the pathway “Microbial metabolism in diverse environments” includes at least five discriminant reactions. Additionally, a small number of reactions related to the nitrogen cycle and to the nitrogen metabolism are detected. Finally, pathways such as “Histidine metabolism” and “Amino sugar and nucleotide sugar metabolism” are also detected within this group.

A substantial number of KEGG pathways specifically characterize the EI cluster. Most of the reactions distinguishing EI from AA are associated with KEGG pathways such as “Microbial metabolism in diverse environments”, “Biosynthesis of secondary metabolites” and “Metabolism of xenobiotics by cytochrome P450”, each comprising more than 20 discriminant reactions. Additional pathways, including “Steroid hormone biosynthesis” and “Biosynthesis of cofactors,” are also highly relevant for characterizing the EI cluster.

Several pathways related to fatty acid metabolism—including “Biosynthesis of unsaturated fatty acids” and “Butanoate metabolism”, which are involved in the production of short-chain fatty acids (SCFAs)—further characterize this cluster and reflect the microbial capacity to transform dietary and endogenous substrates into bioactive lipids. In contrast, the “Benzoate metabolism” pathway involves the degradation of aromatic compounds, highlighting the microbial community’s ability to process dietary and endogenous phenolic compounds.

#### 3.4.2. Discriminative Analysis Between Groups AA and AE

**Figure 11:**
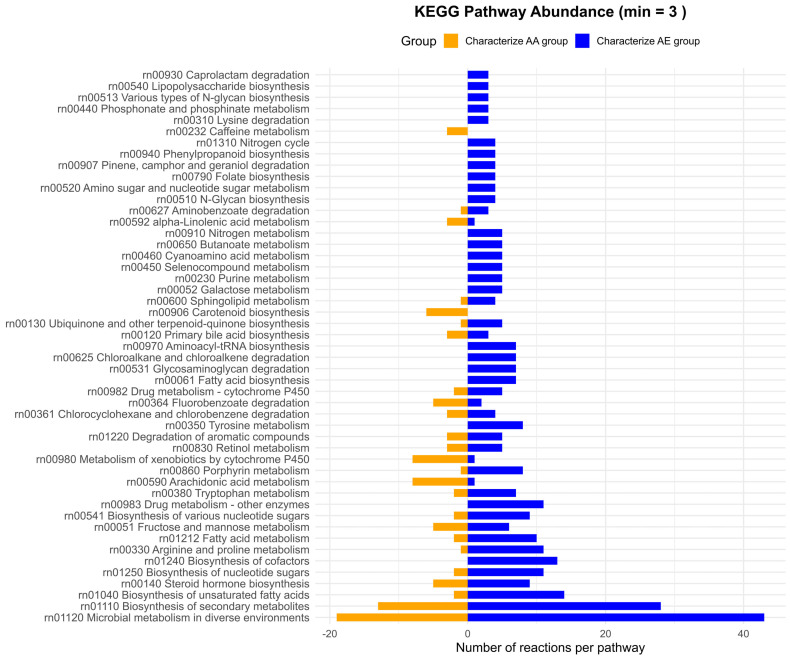
Pathwaylevel distribution of reactions discriminating between clusters AA and AE. Bar plots show the number of reactions assigned to KEGG pathways that characterize the AA cluster (left, orange) and the AE cluster (right, blue).

As shown in Figure 11, relatively few reactions and pathways characterize the AA cluster compared with the AE cluster. However, certain pathways are moderately represented, such as “Microbial metabolism in diverse environments” and “Biosynthesis of secondary metabolites”. In addition, there is a notable presence of pathways with relevant representation among the discriminative features, including “Metabolism of xenobiotics by cytochrome P450”, “Steroid hormone biosynthesis”, or “Arachidonic acid metabolism”. It is also noteworthy that pathways related to the metabolism of the amino acid tryptophan and “Carotenoid biosynthesis” were detected, and may warrant further investigation.

In contrast, the AE cluster is characterized by a high number of reactions, particularly from pathways such as “Microbial metabolism in diverse environments”, “Biosynthesis of secondary metabolites”, “Biosynthesis of unsaturated fatty acids”, or “Biosynthesis of cofactors”. In addition, a significant number of reactions are involved in amino acid metabolism, including “Tryptophan metabolism” and “Tyrosine metabolism”.

#### 3.4.3. Discriminative Analysis Between Groups AE and EI

**Figure 12:**
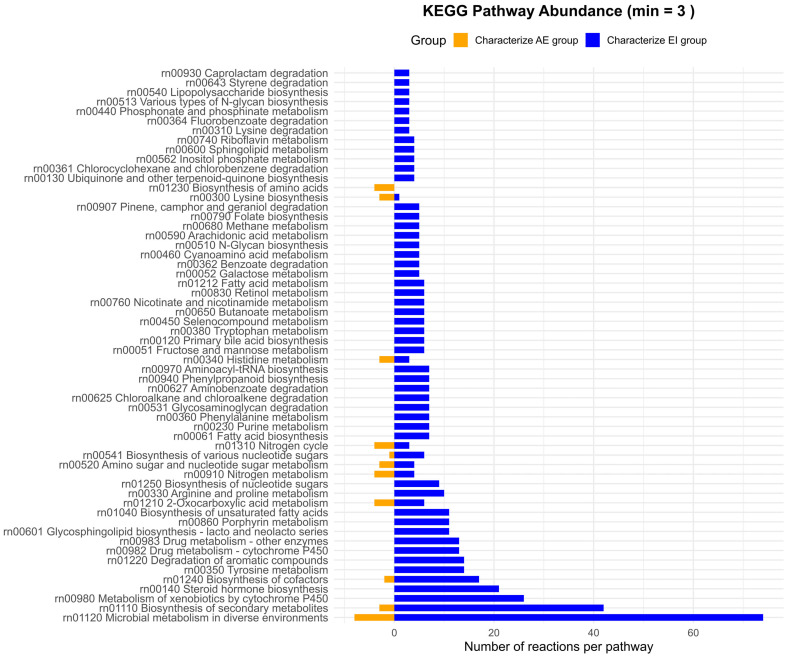
Pathway-level distribution of reactions discriminating between clusters AE and EI. Bar plots show the number of reactions assigned to KEGG pathways that characterize the AE cluster (left, orange) and the EI cluster (right, blue).

As in the AA vs. EI comparison (Figure 12), the number of reactions and pathways characterizing the AE cluster is generally low. However, certain pathways are moderately represented, including “Microbial metabolism in diverse environments” and “Biosynthesis of secondary metabolites”. In addition, there is a notable presence of pathways with relevant representation among the set of discriminative features, such as “Metabolism of xenobiotics by cytochrome P450”, “Steroid hormone biosynthesis”, and “Biosynthesis of cofactors”. It is also noteworthy that pathways related to the metabolism of non-aromatic amino acids (arginine and proline) and aromatic amino acids (tyrosine and tryptophan) are also detected.

In contrast, the EI cluster is characterized by a high number of reactions, particularly within the pathways “Microbial metabolism in diverse environments”, “Biosynthesis of secondary metabolites”, and “Metabolism of xenobiotics by cytochrome P450”. Additional pathways contributing to this profile include “Steroid hormones biosynthesis”, “Biosynthesis of cofactors”, “Tyrosine metabolism”, and “Degradation of aromatic compounds”.

## 4. Discussion

The human gut microbiota plays a central role in host physiology, influencing digestion, immune function, and metabolic homeostasis. Understanding how gut microbiota metabolism changes across life stages can provide insight into age-related physiological processes [42,43]. In this work, we explored the functional organization of gut microbiota metabolism across distinct age groups using a network-based framework grounded in metabolic Directed Acyclic Graphs (m-DAGs). While previous studies have documented age-associated differences in microbiome composition and functional potential, our results demonstrate that such differences are also reflected in the topological organization of community metabolic networks. As this study is observational, the reported age-associated patterns in metabolic network structure do not imply causality, but instead highlight candidate pathways and structural features that may warrant further experimental investigation.

### 4.1. Network-Based Representations of Community Metabolism

Genome-scale metabolic reconstructions have long been recognized as a powerful tool to investigate microbial metabolism beyond descriptive annotations. Large-scale efforts such as the AGORA and AGORA2 resources [44,45] have enabled systematic analyses of metabolic capabilities and interactions among members of the human gut microbiota. However, translating this detailed metabolic information into comparative, sample-level analyses remains challenging, particularly when relying on pathway abundance profiles that overlook the structural organization and interdependencies of metabolic reactions.

The m-DAG framework addresses this limitation by embedding enzymatic repertoires into coherent network structures. By collapsing strongly connected components into MBBs, m-DAGs capture cyclic dependencies and functional modules that likely operate as integrated units in vivo. Unlike predefined pathway annotations, MBBs emerge directly from network topology, reflecting functional modules defined by reaction inter-dependencies rather than curated pathway classifications. This abstraction preserves essential metabolic relationships while reducing network complexity, enabling systematic comparisons across samples and groups.

### 4.2. Topological Signatures of Age-Associated Metabolic Organization

Topological analysis revealed marked differences in metabolic network organization across infants, adults, and elderly individuals. Infant networks consistently exhibited larger sizes, with higher numbers of reactions, enzymes, compounds, and MBBs, along with relatively homogeneous topological properties across samples. Adult networks, in contrast, were more compact, with fewer MBBs and higher edge density, and exhibited greater inter-individual variability. Elderly networks showed pronounced heterogeneity, spanning configurations similar to both infant-like and adult-like profiles.

Previous studies have reported age-associated differences in microbiome function, particularly during early life and late adulthood [42,46]. However, these studies typically quantify functional variation as pathway abundance or metabolite concentrations. Our results demonstrate that such differences are also encoded in the global architecture of metabolic networks, emphasizing age-associated modes of functional organization rather than differences in functional content alone.

### 4.3. Functional Contraction, Redundancy, and Robustness

Adult m-DAGs were characterized by a reduced number of MBBs and reactions, suggesting a contraction of functional space relative to infancy. At the same time, higher edge density and a lower proportion of essential MBBs indicate a compact yet relatively robust organization, in which metabolic functions are tightly interconnected and less sensitive to the removal of individual modules. This pattern is consistent with reports describing functional stabilization and consolidation in adult gut microbiomes [15,47].

In contrast, infant networks exhibited broader functional repertoires and greater uniformity in dependence on specific MBBs, potentially reflecting a developmental stage in which metabolic versatility is required to accommodate rapid dietary and physiological changes. Elderly networks again showed mixed patterns, reinforcing the view that aging is associated with diverse functional configurations rather than a single, unidirectional decline.

### 4.4. Heterogeneity of Elderly Networks and Alternative Functional States

One of the most notable findings of this study is the bifurcation observed within the elderly cohort (in the AE cluster and the IE cluster). At the same time, some elderly individuals displayed adult-like, compact metabolic networks, while others clustered with infant-like profiles characterized by larger, more interconnected architectures. This observation aligns with previous reports describing metabolic heterogeneity among elderly populations and the persistence of microbial features associated with metabolic health in subsets of older individuals [48,49].

Rather than reflecting a simple loss of metabolic functions, our network-based results suggest that aging may involve reorganization and redistribution of metabolic capabilities, with alternative functional states coexisting within the same age group. Importantly, these patterns emerge directly from network structure, independently of taxonomic composition.

### 4.5. Network Dissimilarities Reveal Latent Functional Groupings

By quantifying pairwise dissimilarities between m-DAGs using an MBB-based matching approach, we identified functional clusters that did not strictly correspond to chronological age. Instead, samples were grouped according to shared metabolic architectures, with one cluster dominated by adult samples and another grouping infants together with a subset of elderly individuals.

This finding extends previous observations that functional beta-diversity can uncover patterns not captured by taxonomic comparisons alone [50]. Moreover, the consistently lower intra-individual dissimilarity observed among infant samples suggests that network-level metabolic organization represents a relatively stable individual trait during early life, at least within the temporal resolution examined.

### 4.6. Discriminant Metabolic Building Blocks as Mechanistic Links

The identification of discriminant MBBs and reactions provides a mechanistic link between global network patterns and specific metabolic processes. Pathways related to carbohydrate metabolism, fermentation, lipid metabolism, and xenobiotic processes were prominent in networks clustering with infant-like and EI profiles, consistent with the known importance of microbial metabolites such as short-chain fatty acids in early life [51]. Conversely, adult-like clusters were characterized by networks dominated by core metabolic pathways, reflecting consolidation around central metabolic functions.

By enabling network-level differences to be traced back to specific enzymatic modules and pathways, the m-DAG framework supports biologically interpretable hypotheses that go beyond descriptive comparisons of pathway abundance.

## 5. Conclusions

In this study, we demonstrate that network-based representations of microbiome metabolism provide a robust framework for characterizing functional organization across the human lifespan. By reconstructing community metabolic networks and integrating genome-scale metabolic knowledge into m-DAGs, we show that age-associated differences in gut microbiota metabolism are reflected not only in functional content but also in the topological structure of metabolic networks.

Our results indicate that adult gut microbiota metabolism is characterized by a compact and relatively robust network architecture. In contrast, infants and a subset of elderly individuals exhibit broader, more interconnected metabolic repertoires. Importantly, functional similarity among samples does not strictly follow chronological age, revealing latent metabolic states that transcend conventional age categories.

Overall, this work highlights the added value of network-centric approaches for microbiome research, enabling the integration of genome-scale metabolic information into interpretable structural descriptors. The m-DAG framework offers a scalable strategy for exploring functional organization in microbial communities. It establishes a foundation for further studies linking metabolic network architecture to host physiology, health, and disease.

## Figures and Tables

**Table 1:** Samples included in each cluster identified by hierarchical clustering using Munkres dissimilarity.

Group	Individual	Samples	Number of Samples
AA	A01	A01T6 A01T7 A01T8	3
	A02	A02T1 A02T4 A02T5 A02T6 A02T7 A02T8	6
	A03	A03T1 A03T4 A03T5 A03T6 A03T7	5
	A04	A04T1	1
AE	A04	A04T4 A04T5 A04T6 A04T7 A04T8	5
	A05	A05T1 A05T4 A05T5 A05T6 A05T7 A05T8	6
	A06	A06T1 A06T4	2
	A07	A07T1 A07T4 A07T5 A07T6 A07T7 A07T8	6
	A08	A08T1 A08T4 A08T5 A08T6 A08T7 A08T8	6
	A09	A09T1 A09T4 A09T5 A09T6 A09T7 A09T8	6
	A10	A10T1 A10T4	2
	E01	E01T1 E01T4 E01T5 E01T6 E01T7 E01T8	6
	E02	E02T1 E02T4 E02T5 E02T6 E02T7 E02T8	6
	E03	E03T1 E03T4 E03T5 E03T6 E03T7 E03T8	6
	E04	E04T4 E04T5 E04T7 E04T8	4
	E05	E05T1 E05T5 E05T6 E05T7	4
AI	E05	E05T8	1
	E06	E06T1 E06T4 E06T5 E06T6 E06T7	5
	E07	E07T1 E07T4 E07T6 E07T7	4
	E09	E09T1 E09T4 E09T5 E09T6 E09T7 E09T8	6
	E10	E10T1 E10T4 E10T5 E10T6 E10T7 E10T8	6
	I01	I01T1 I01T4 I01T5 I01T6 I01T7 I01T8	6
	I02	I02T1 I02T4 I02T5 I02T6 I02T7 I02T8	6
	I03	I03T1 I03T4 I03T5 I03T6 I03T7 I03T8	6
	I04	I04T1	1
	I05	I05T1 I05T4 I05T5 I05T6 I05T7 I05T8	6
	I06	I06T1 I06T4 I06T5 I06T6 I06T7 I06T8	6
	I07	I07T1 I07T4 I07T5 I07T6 I07T7 I07T8	6
	I08	I08T1 I08T4 I08T5 I08T6 I08T7 I08T8	6
	I09	I09T1 I09T4 I09T5 I09T6 I09T7 I09T8	6
	I10	I10T1 I10T4 I10T5 I10T6 I10T7 I10T8	6

**Table 2:** Key characteristics of core and pan m-DAGs for groups defined by age, Munkres dissimilarity, and the entire cohort (after exclusion of outliers). Individual-level data for all samples are available in Table S1.

Prop. Essential Nodes	Essential MBBs	MBBs Single Reaction	MBBs More 1 Reaction	Total No. MBBs	No. Compounds	No. Enzymes	No. Reactions	
0.1364	111	681	133	814	2548	1435	4283	AA core
0.1565	161	890	139	1029	3095	1856	5501	AA pan
0.1607	172	930	140	1070	3150	1885	5591	AE core
0.1510	174	1012	140	1152	3394	2084	6125	AE pan
0.1562	191	1081	142	1223	3493	2120	6322	EI core
0.1611	202	1111	143	1254	3557	2171	6459	EI pan
0.1364	111	681	133	814	2548	1435	4283	Adults core
0.1613	182	982	146	1128	3304	2011	5945	Adults pan
0.1612	182	984	145	1129	3307	2014	5949	Elders core
0.1542	190	1089	143	1232	3503	2135	6352	Elders pan
0.1550	191	1089	143	1232	3503	2135	6352	Infants core
0.1611	202	1111	143	1254	3557	2171	6459	Infants pan
0.1364	111	681	133	814	2548	1435	4283	Total core
0.1603	201	1111	143	1254	3557	2171	6459	Total pan

## Data Availability

The mDAG reconstruction experiments can be accessed at the following links: https://bioinfo.uib.es/metadag/handleExperiment/df568f6d-5ee6-3f04-8732-a6d1f167de20 (accessed on 8 February 2026); https://bioinfo.uib.es/metadag/handleExperiment/5b4c005b-529a-3b57-bf45-7fe8beaf290f (accessed on 8 February 2026).

## Supplementary Materials

The supporting information cited in text can be downloaded at: https://www.mdpi.com/article/10.3390/metabo16020140/s1. Table S1: Key characteristics of mDAGs by individual; Table S2: Topological descriptors of mDAGs by individual.
